# Optic nerve sheath measurement to monitor disease activity in giant cell arteritis: a pilot study

**DOI:** 10.1007/s10067-024-07095-z

**Published:** 2024-08-07

**Authors:** Carolyn Ross, Stéphanie Ducharme-Bénard, Samer Hussein, Rosalie-Sélène Meunier, Christian Pagnoux, Jean-Paul Makhzoum

**Affiliations:** 1 Vasculitis Clinic, Department of Medicine, Sacre-Coeur Hospital, University of Montreal, Montreal, QC Canada; 2 Vasculitis Clinic, Department of Medicine, Mount Sinai Hospital, University of Toronto, Toronto, ON Canada

**Keywords:** Biomarkers, Diagnosis, Giant cell arteritis, Optic nerve, Ultrasonography

## Abstract

**Introduction/Objectives:**

Optic nerve sheath (ONS) enhancement using magnetic resonance imaging of the orbits was observed in patients with giant cell arteritis (GCA). We previously showed that ONS diameter (ONSD) by bedside ultrasound is increased in patient with active GCA. This study aims to assess whether ONSD decreases with clinical remission in patients with GCA.

**Methods:**

A prospective cohort study was conducted from June 2022 to January 2023. Patients who had an optic nerve ultrasound at GCA diagnosis as part of a previous crosssectional study were eligible. Optic nerve ultrasound was performed by the same investigator at diagnosis and month 3. ONSD (includes the optic nerve and its sheath) and optic nerve diameter (OND) were measured. Descriptive statistics for baseline characteristics and paired sample t-test were performed to assess the mean difference in OND and ONSD between diagnosis and month 3.

**Results:**

Nine patients with GCA were included. The median age at disease onset was 79 years (interquartile range (IQR) of 79–82 years), and 7 patients were males. All patients were in clinical remission at month 3 on prednisone (median dose of 15 mg/day, IQR of 10–25 mg). The mean ONSD was lower at month 3 (3.76 mm) compared to baseline (5.98 mm), with a paired mean difference of 2.22 mm (95% CI 1.41–3.03 mm, *p* < 0.001). As anticipated, OND measurements did not vary between diagnosis and month 3.

**Conclusion:**

ONSD on ultrasound improves after 3 months of therapy in patients with GCA. A longer prospective study is required to determine if ONSD is useful to assess disease activity in GCA.

**Table:** 

**Key Points**
• *ONS ultrasound can identify patients with active GCA.* • *The ONSD on ultrasound is dynamic and improved after 3 months of GCA therapy.* • *ONS ultrasound may be useful to monitor disease activity in GCA.*

## Introduction

Giant cell arteritis (GCA) affects adults over the age of 50 years and can cause a wide array of symptoms, such as headaches, ischemic visual changes, jaw claudication, stroke, and polymyalgia rheumatica [1]. One of the most feared complications of GCA is permanent visual impairment, reported in 15–30% of patients [2, 3]. Inflammation affecting the posterior ciliary arteries, branches of the ophthalmic artery, and primary blood supply to the anterior optic nerve head can cause anterior ischemic optic neuropathy, which is the leading cause of vision loss in GCA [2, 4].

Despite newer treatments such as biologics blocking interleukin 6 (IL-6), GCA relapses are common [5]. We often rely on the C-reactive protein (CRP) and erythrocyte sedimentation rate (ESR) to monitor disease activity. However, inflammatory markers are not specific to GCA and can be elevated in many conditions. Disease relapses can also be difficult to ascertain when the CRP and ESR remain normal. This is the case with tocilizumab, which normalizes the CRP and ESR [6–8]. Thus, there is a clear unmet need for better tools to monitor disease activity in GCA [6].

The use of non-invasive imaging modalities in the diagnosis of GCA has steadily increased over the last years. Color doppler ultrasound (CDUS) of temporal/axillary arteries is widely used in Europe and is proven to have a high sensitivity (Se > 90%) and specificity (Sp > 90%) [9, 10]. In most countries, CDUS of temporal/axillary arteries is not widely available and is operator- and equipment-dependent [11]. Several studies have described the involvement of the ophthalmic and posterior ciliary arteries, as well as optic nerve sheath enhancement on contrast-enhanced magnetic resonance imaging (MRI) of the orbits and brain in patients with GCA presenting with ischemic optic neuropathy. These findings have also been reported in GCA patients unaffected by visual symptoms [12–19].

**Fig. 1:**
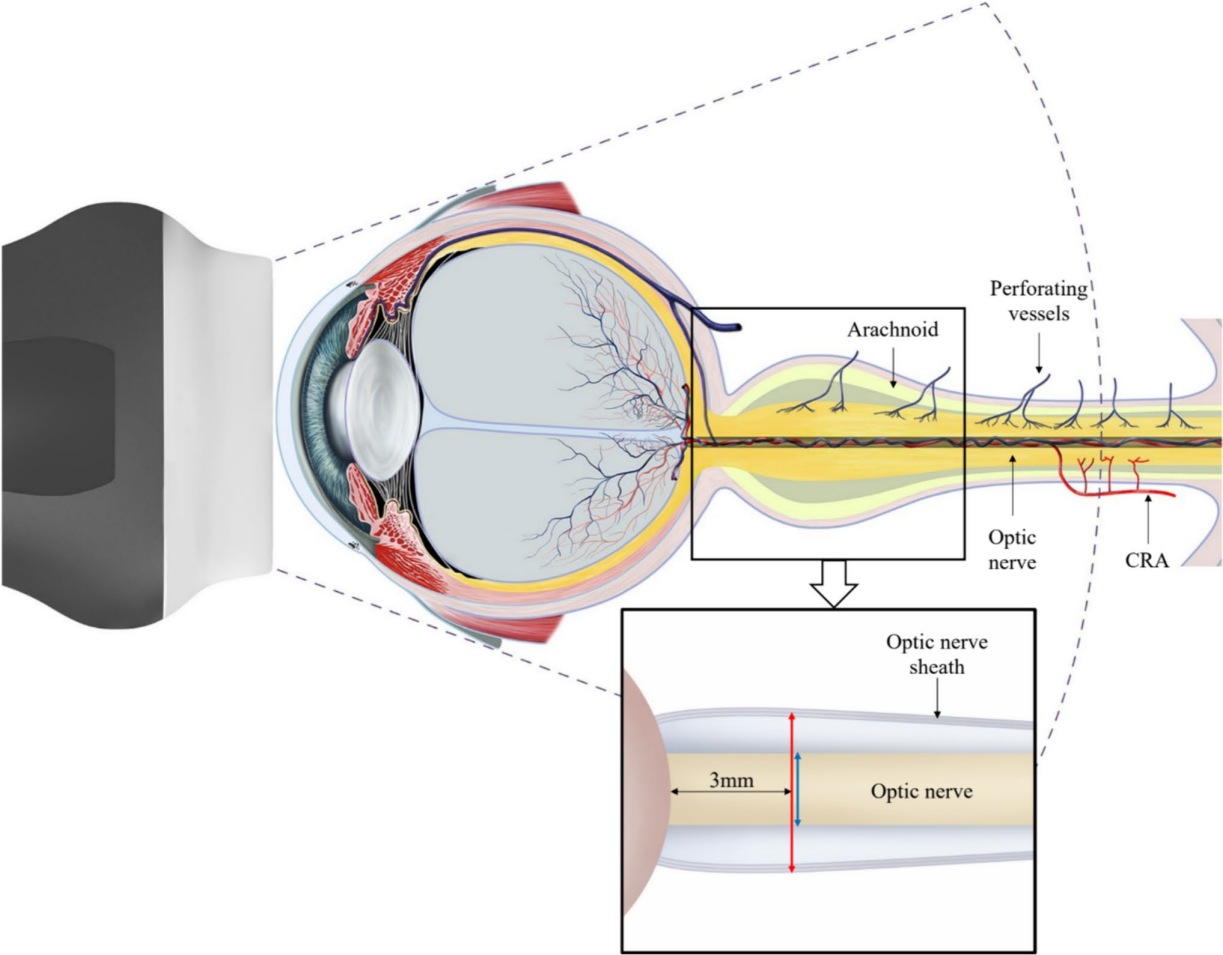
Optic nerve sheath anatomy and measurements performed on optic nerve ultrasound. Blue arrow: optic nerve diameter (OND). Red arrow, optic nerve sheath diameter (ONSD). CRA, central retinal artery

We previously performed a diagnostic test accuracy study using optic nerve ultrasound as a novel, bedside, rapid GCA diagnostic tool. We demonstrated that optic nerve sheath diameter (ONSD) is significantly greater in patients with new-onset, active GCA when compared to controls [20]. We hypothesized that this finding is likely due to an inflammatory involvement of pial arteries supplying the nerve sheath, causing optic perineuritis (Fig. 1) [21]. Whether this large ONSD persists or improves with GCA therapy is unknown. Using optic nerve ultrasound as a method to monitor disease activity in GCA has never been done. The objective of this study is to assess ONSD changes when clinical remission is achieved in patients with GCA.

## Materials and methods

### Study design

We conducted a prospective inception cohort study at our tertiary vasculitis clinic in Montreal (Canada), part of the Canadian Vasculitis Research Network (CanVasc), from June 1, 2022, to January 1, 2023. The follow-up period was 6 months after inclusion.

### Participants

Consecutive participants who had an ON ultrasound at GCA diagnosis as part of a previous cross-sectional, diagnostic test accuracy study were approached and offered participation in this study. All patients previously had a baseline CDUS of temporal/axillary artery, which is the standard GCA assessment procedure in our clinic. In this initial diagnostic test accuracy study, participants were included if they were adults > 18 years of age, referred for clinically suspected new-onset GCA, had received < 14 days of systemic glucocorticoids (orally or intravenously), and had no known history of intracranial hypertension or retinal or demyelinating disease [20].

Participants had to meet the following criteria to be included in this GCA inception cohort: (1) fulfill the 2022 American College of Rheumatology (ACR)/European Alliance of Associations for Rheumatology (EULAR) classification criteria for GCA [22], (2) have unequivocal signs of GCA on CDUS of temporal/axillary arteries, (3) have had successful completion of optic nerve ultrasound at the time of GCA diagnosis as part of a previous cross-sectional study [20], and (4) acceptance to comply with study visits. Participants who did not meet these criteria or refuse to participate were excluded.

### Study visits and procedures

For each patient, we recorded a baseline visit at GCA diagnosis (month 0) and a follow-up visit (month 3). Standardized data on demographics, past medical history, medications, clinical manifestations of GCA, physical exam, and blood work were collected at each visit. A CDUS of temporal and axillary arteries was also performed at each visit (Canon Xario™ 200 Platinum series with an 18L7 probe for cranial arteries and a 14L5 probe for axillary arteries). Ultrasonographers were experienced physicians in running GCA ultrasound fast-track clinics (SDB and JPM, both with > 7 years of experience and > 1500 exams performed) and previously published temporal/axillary CDUS validation cohorts [9, 23].

Optic nerve ultrasound was performed at each visit (month 0 and month 3) by the same investigator, without blinding to clinical information. Optic nerve ultrasound measures at month 3 were performed without consulting the initial baseline images or measures. Patients were examined in a supine position. The probe was gently placed on the closed eyelid and adjusted to a suitable angle to display the optic nerve entry into the eyeball, with a 14L5 probe with a limit of thermal index ≤ 1, mechanical index ≤ 0.23 and an intensity limit of ≤ 50 mW/cm^2^. Ultrasound measurements were performed for both eyes 3 mm distal to the posterior aspect of the ocular globe.

### Outcome measures

Optic nerve sheath diameter (ONSD) and optic nerve diameter (OND) were measured. ONSD consists of the measurement of the optic nerve and its sheath, whereas OND is the measurement of the optic nerve without its sheath (Fig. 1). The upper limit of normal of ONSD is unknown in patients with GCA, but is up to 5 mm in young, healthy adults according to emergency and intensive care literature [23–25]. We considered a normal threshold of ONSD as ≤ 5 mm in healthy adults. These measures were recorded for each eye at the baseline visit (month 0) and at month 3.

### Study definitions

The final confirmation of GCA involved a two-step, composite clinical reference standard. At study inclusion, participants had to meet the 2022 ACR/EULAR classification criteria for GCA and have unequivocal signs of GCA on color Doppler ultrasound of temporal/axillary arteries. In addition, GCA was confirmed 6 months later by a vasculitis expert based on the initial clinical presentation and on the evolution of signs, symptoms, and test results.

Clinical remission was defined as the absence of signs and symptoms attributable to active GCA. Disease relapse was a reoccurrence of signs and symptoms due to active GCA after achieving clinical remission and/or abnormal inflammatory markers leading to an increase or change in treatment. Clinical persistent vision defect was defined as unilateral or bilateral partial or complete vision loss persisting at month 3.

### Statistical analysis

Descriptive statistics were used to describe characteristics of participants, with means and standard deviations (SD), or medians and interquartile ranges, according to the data distribution. Mean OND and ONSD and their SD were calculated for each eye, at baseline and month 3. The mean OND and ONSD (using both eyes) was also calculated, for each participant, at baseline and month 3.

Paired sample *t*-test was used to compare mean OND and ONSD from the baseline visit to month 3. A two-tailed significance level of 0.05 was used. Statistical analyses were performed using StataSE (StataCorp, V17.0).

### Sample size

In our initial, cross-sectional, diagnostic accuracy study, the standard deviation for ONSD in patients with active GCA was 1.4 mm. In published studies measuring ONSD before and after lumbar puncture for idiopathic intracranial hypertension, ONSD measurements improved between 0.5 and 2.9 mm after procedure [26]. We aimed to detect a reduction of 1.5 mm in ONSD in participants with GCA after 3 months of therapy, with a power of 0.8 and a type 1 error of 0.05. A sample size of nine participants was required for this paired analysis.

### Ethics

This study was approved by our institution’s research ethics board (protocol number 20201890). All patients consented to be enrolled in this study and provided informed signed consent. The study was conducted in accordance with the Declaration of Helsinki (2013 revision).

## Results

Nine patients with new-onset active GCA were included in this study. All patients were in clinical remission at month 3. Their main demographics and baseline characteristics are listed in Table 1. Median age at diagnosis was 79 years (IQR 79–82 years) and 7 patients were males.

**Table 1:** Baseline characteristics of nine patients with new-onset GCA

Baseline characteristics	Number of patients (%)
Age (years), median (IQR)	79 (79–82)
Female, *n* (%)	2 (22)
Caucasian ethnicity, *n* (%)	9 (100)
Smoking status, *n* (%)	
Previous smoker	5 (55)
Active smoker	2 (22)
Cardiovascular risk factors, *n* (%)	
CAD	3 (33)
HTN	5 (55)
Dyslipidemia	4 (44)
Diabetes	2 (22)
PAD	1 (11)

*GCA* giant cell arteritis, *IQR* interquartile range, *n* number of patients, *CAD* coronary artery disease, *HTN* hypertension, *PAD* peripheral artery disease

GCA manifestations and therapy at the baseline visit (month 0) and at month 3 are presented in Table 2. Headaches were present in 78% of participants at baseline and had resolved in all patients at month 3. Four patients had vision loss at diagnosis, all with anterior ischemic optic neuropathy on retinal exam. Only three patients had persistent vision loss at month 3, whereas one patient had partial recovery of visual function. Five patients had abnormal temporal arteries on physical exam at diagnosis. The median CRP was 60 mg/L (IQR 41–88) at baseline and 4 mg/L (IQR 3–23) at follow-up (month 3). The median duration of glucocorticoid treatment before the initial assessment was 5 days (IQR 2–6), and the median dose of glucocorticoids (prednisone equivalent) was 50 mg (IQR 50–50) at the baseline visit, and 15 mg (IQR 10–25) at month 3. In terms of adjunctive therapies, five patients were treated with tocilizumab and one patient with leflunomide.

**Table 2:** Clinical features and treatments at baseline visit (diagnosis of GCA) and month 3 (clinical remission) of nine patients with new-onset GCA

GCA features	Baseline visit (month 0)	Follow-up (month 3)
Symptoms, *n* (%)		
Headache	7 (78)	0
Scalp tenderness	4 (44)	0
Constitutional symptoms	4 (44)	1 (11)
Jaw claudication	5 (56)	0
PMR	3 (33)	0
Diplopia	1 (11)	0
Amaurosis fugax	1 (11)	0
Complete vision loss	4 (44)	3 (33)
Physical examination, *n* (%)		
Abnormal temporal artery exam	5 (56)	0
AION	4 (67)^a^	N/A
Inflammatory markers, median (IQR)		
C-reactive protein (mg/L)	60 (41–88)	4 (3–23)
ESR (mm/h)	62.5 (58–64)	21 (13–32)
GC dosage (prednisone equivalent)		
Days on GC prior to assessment, median (IQR)	5 (2–6)	106 (98–107)
GC dose at assessment (mg), median (IQR)	50 (50–50)	15 (10–25)
Intravenous methylprednisolone, *n* (%)	5 (56)	N/A
Immunosuppressive therapies, *n* (%)		
Tocilizumab	0	5 (56)
Methotrexate	0	0
Leflunomide	0	1 (11)

*GCA* giant cell arteritis, *n* number of patients, *PMR* polymyalgia rheumatica, *AION* anterior ischemic optic neuropathy, *N/A* not applicable, *IQR* interquartile range, *ESR* erythrocyte sedimentation rate, *GC* glucocorticoid

^a^Ophthalmology exam was performed in 6 patients with GCA

### Comparison of optic nerve measurements between baseline and month 3 visits

The mean ONSD for the right eye was 6.10 mm (SD 1.16 mm) at diagnosis and 4.14 mm (SD 1.14 mm) at month 3 (Table 3). For the left eye, the mean ONSD was 5.86 mm (SD 1.38 mm) at diagnosis and 3.38 mm (SD 0.44 mm) at month 3. Mean ONSD was lower at month 3 (3.76 mm) compared to baseline (5.98 mm), with a paired mean difference of 2.22 mm (95% CI 1.41–3.03 mm, *p* < 0.001). There was no significant difference in mean OND between the baseline visit (2.97 mm) and month 3 (2.63 mm), with a paired mean difference of 0.34 mm (95% CI − 0.10 to 0.78 mm, *p* = 0.11).

**Table 3:** Comparison of optic nerve measurements on ultrasound at baseline visit (diagnosis of GCA) and month 3 (clinical remission) in nine patients with new-onset GCA

Measurements (in mm), mean (SD)	Baseline visit (month 0)	Follow-up (month 3)	Mean difference (month 0–month 3)	95% CI	*P* value
Paired analysis of participants					
ONSD^a^	5.98 (1.17)	3.76 (0.60)	2.22	1.41 to 3.03	< 0.001
OND^a^	2.97 (0.46)	2.63 (0.38)	0.34	− 0.10 to 0.78	0.11
Assessment for each eye					
Right ONSD	6.10 (1.16)	4.14 (1.14)	1.96	1.20 to 2.71	–
Left ONSD	5.86 (1.38)	3.38 (0.44)	2.48	1.39 to 3.56	–
Right OND	3.22 (0.35)	2.90 (0.66)	0.32	− 0.06 to 0.70	–
Left OND	2.72 (0.76)	2.37 (0.43)	0.36	− 0.33 to 1.04	–

*GCA* giant cell arteritis, *mm* millimeters, *CI* confidence interval, *SD* standard deviation, *ONSD* optic nerve sheath diameter, *OND* optic nerve diameter

^a^Mean ONSD and OND were computed for each participant using measures obtained for both eyes

**Fig. 2:**
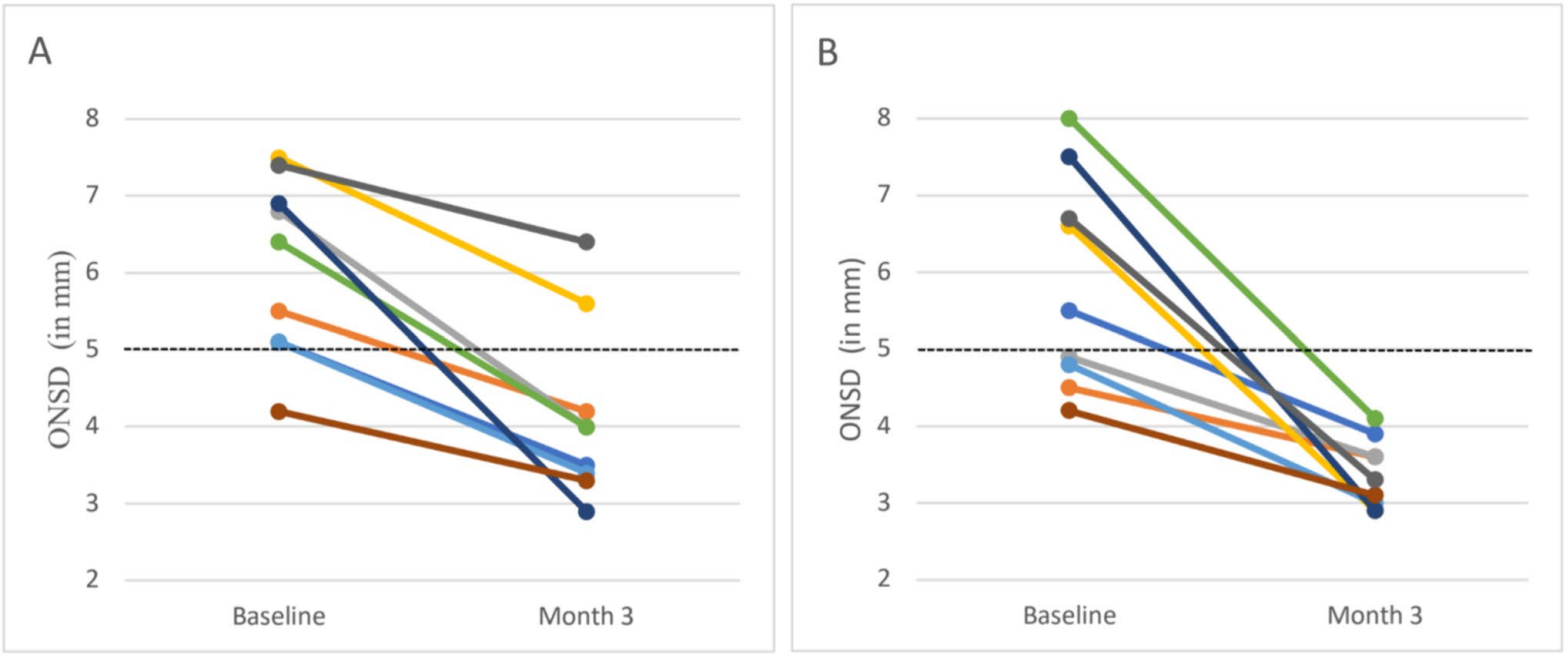
Evolution of ONSD on ultrasound from month 0 (active GCA) to month 3 (clinical remission) in nine patients with new-onset GCA. **A** ONSD evolution for the right eye in nine participants with GCA. **B** ONSD evolution for the left eye in nine participants with GCA. Dotted line: threshold of ONSD ≤ 5 mm is considered normal in healthy young adults. ONSD, optic nerve sheath diameter; GCA, giant cell arteritis

The decrease in ONSD, for each participant, over time is presented in Fig. 2. All patients at month 3 had an ONSD below the upper limit of normal for healthy, young adults (≤ 5 mm) in at least one eye. Two participants still had an ONSD above 5 mm, in one eye only, at month 3. These patients had the largest ONSD at the baseline visit and still improved at month 3 (a decrease in ONSD from 7.5 to 5.6 mm for one participant, and from 7.4 to 6.4 mm in the second participant).

In participants with persistent vision loss during the follow-up, the mean ONSD was 5.33 mm (SD 1.66 mm) at baseline and 3.78 mm (SD 0.53 mm) at month 3. Similarly, participants without visual symptoms at follow-up had a mean ONSD of 5.23 mm (SD 2.48 mm) at baseline and 3.75 mm (SD 0.68 mm) at month 3.

## Discussion

We found that ONSD, measured with optic nerve ultrasound, decreased after 3 months of therapy in patients with newly diagnosed GCA. We had also previously demonstrated that ONSD is greater in patients with new-onset, active GCA, compared to patients without GCA [20]. Therefore, optic nerve ultrasound may be useful not only to detect GCA, but also to assess disease activity over time. To our knowledge, this is the first study to assess optic nerve ultrasound measurements as a tool to monitor disease activity in GCA.

In critical care literature, ONSD below 5 mm is considered normal in healthy young adults [27]. It is unknown if the same cut points can be applied in GCA, because ONSD may be affected by increasing age, sex, and other ophthalmic conditions [28]. In our study, only one participant had an ONSD below 5 mm for both eyes at the baseline visit (at GCA diagnosis). At month 3, all participants were in clinical remission and had an ONSD below 5 mm in at least one eye. The two participants with the greatest ONSD in the right eye at baseline still had an ONSD above 5 mm at month 3. Nevertheless, ONSD still improved with therapy, as in all other patients in clinical remission.

The role of neuroimaging to detect orbital involvement in GCA is promising. Orbital MRI or optic nerve ultrasound can evaluate ocular anatomy posterior to the ocular globe, which is not assessed by standard ophthalmologic exam. A study showed that combining orbital with cranial vessel wall MRI improved diagnostic accuracy when compared with temporal artery biopsy or cranial MRI alone. MRI enhancement of orbital structures improved with treatment over time [29]. Our study also supports that ONSD improves with treatment in patients with clinical remission.

So far, there has been no clear association between optic nerve sheath inflammation and visual symptoms. In a study of 56 patients with GCA, 23% of patients showed enhancement of the optic nerve sheath on MRI, regardless of the presence or absence of ophthalmologic symptoms [2]. We also previously shown that ONSD on ultrasound was similar at diagnosis in GCA patients with and without anterior ischemic optic neuropathy. In this study, ONSD was similar at month 3 in patients with and without persistent visual defect. Whether this represents early ischemic changes and poorer prognostic in a subset of patients with GCA remains unknown.

Our study had several strengths. Our study design was prospective, with systematic, standardized data collection and study procedures. All our patients satisfied the 2022 ACR/EULAR classification criteria, and our study definitions of clinical remission were similar to those commonly used in major clinical trials in GCA [7, 30, 31]. We used well established and reproducible optic nerve ultrasound technique and settings, widely described in the emergency and intensive care literature [32].

There are some limitations to our study. Because ONSD may be subjective and operator-dependent, intraclass and interclass correlation needs to be assessed in a subsequent study. All participants were in clinical remission at month 3. We were thus unable to compare the ONSD evolution (from baseline to month 3) in patients in remission and in those with active disease. Because this was a pilot study of a novel exam in GCA, we calculated the sample size based on a clinically significant detectable difference in ONSD. The small sample size did not allow hypothesis testing in subgroups, such as comparing patients with and without persistent vision defect at month 3. Nevertheless, this is the first study to introduce the concept of ONSD monitoring on ultrasound. This pilot study will inform the design of a larger and longer prospective trial. The investigators were not blinded to clinical symptoms, leading to a potential overestimation of the performance of this exam. However, optic nerve ultrasound at month 3 was performed without consulting the baseline measurements (at diagnosis) of OND and ONSD.

Positivity cutoff values of ONSD in GCA remain unknown and will need to be defined through a larger diagnostic accuracy study with a sufficient sample size to maximize precision in ultrasound measurements. This study demonstrated a decrease in ONSD on ultrasound in GCA patients in clinical remission; whether ONSD re-increases with a subsequent GCA relapse is unknown and requires further investigation.

Optic nerve ultrasound is a non-invasive, bedside, and easy-to-learn technique. It can be performed in less than 5 min and does not require sophisticated equipment. In fact, optic nerve ultrasound has been integrated in many medical residency training programs due to its usefulness in the critical care setting for detecting signs of intracranial hypertension. Accurate ONSD measurement may also be possible using pocket-sized ultrasound devices despite their lower resolution [32]. Therefore, bedside optic nerve ultrasound has several advantages in terms of accessibility and cost compared to orbital MRI. Although more research is still required, integrating bedside optic nerve ultrasound in a regular GCA disease activity assessment would be easy and feasible. ONSD on ultrasound may potentially be useful to monitor disease activity in GCA. This could potentially facilitate the follow-up of GCA patients with nonspecific symptoms and normal CRP due to tocilizumab.

## Conclusion

We found that ONSD measured on optic nerve ultrasound improved with therapy between the baseline visit (GCA diagnosis) and at month 3 (clinical remission) in patients with active, new-onset GCA. Thus, ONSD on ultrasound may potentially be a tool to monitor disease activity in GCA. A larger, longer prospective trial is planned to confirm these findings and assess if ONSD re-increases with GCA relapses.

## Data Availability

The deidentified data that support the findings of this study are available from the corresponding author, JPM, after review and approval of a research proposal and statistical analysis plan and execution of a data-sharing agreement. Data requests can be submitted at any time, and the data will be accessible for 12 months.
